# Cooperative protection of technical secrets in cultural industry cluster-based on evolutionary game model of leading enterprises and following enterprises

**DOI:** 10.1371/journal.pone.0291459

**Published:** 2023-09-14

**Authors:** Mingxia Xu, Zuojiao Hu

**Affiliations:** School of Economics and Management, Zhengzhou University of Light Industry, Zhengzhou, China; Sichuan Agricultural University, CHINA

## Abstract

Due to the confidentiality, value and exclusivity of technical secrets, how to protect the technical secrets within cultural industry clusters has become a paradoxical issue and a research hotspot. Focusing on the collaborative protection of technical secrets within cultural industry clusters, this paper analyzes the strategies of collaborative protection of technical secrets between leading enterprises and following enterprises based on evolutionary game theory, and uses dynamic evolution and simulation methods to identify key factors behind the strategy choices, which further enriches the evolutionary mechanism of collaborative protection, and expands the application scenarios of the theory of evolutionary game. As the research results show, the collaborative protection strategy of technical secrets within cultural industry clusters is feasible, the cost of collaborative protection, government subsidies, and compensation for collaborative deposits are key variables that determine the trend of the game. The government subsidy coefficient, the collaborative deposits, and the difference in the number of technology secrets are strongly sensitive factors underlying the mode of protection, while the synergistic benefits between firms are weakly sensitive factors. Therefore, this paper proposes that increasing government subsidies and collaborative deposits, and reducing the differences in the number of technical secrets among game subjects can promote enterprises to eliminate suspicion and increase their willingness to cooperate in protecting technical secrets.

## 1. Introduction

Under the dual influence of government policies and market mechanisms, cultural industry clusters are gradually becoming powerhouses for economic development and cultural prosperity. The 20th Party Congress emphasizes cultural confidence and self-improvement, and the growth rate of cultural industry is in line with GDP growth in the past three years, which fully demonstrates it is important to develop cultural industry clusters. As a strategic emerging industry with intensive knowledge, flexible cooperation, and cultural creativity [1, 2], cultural industry clusters are hailed as the most promising industry in the 21st century (This paper mainly focuses on the homogeneous type of knowledge-intensive cultural industry clusters). However, just because of these advantages, opportunism runs rampant, and disputes over technical secrets, infringement, and leakage occur frequently. Among them, technical secret is a kind of information technology which is not known to the public and can bring economic benefits to the right holder by virtue of the experience or skill of the enterprise [3], which contains the competitive information of the enterprise such as the product formula, process flow, service know-how and so on. As for the technical secrets of the cultural industry, it not only involves the materialized or technologies capital, production, circulation, and consumption process of creative competition, but also encompasses the dissemination and circulation of culture, spirit, and value, which is the uniqueness of the technical secrets of the cultural industry that distinguishes it from other knowledge-intensive industries [4]. It is because of the hidden nature of the way of existence of the technical secrets of the cultural industry, its own value and uniqueness, which leads to the fact that the enterprises with technical secrets have been in the non-proprietary nature of self-invisible protection, not open and not shared industrial protection system. At the same time, along with the combination of cultural industry and Internet, cultural industry involves "derivatives + IP" cross-domain transactions, also involves script killing, game live and other new types of industry, cultural industry cluster within the classified staff exchanges and cooperation is more intensive, complex, which also aggravate the complexity of the protection of technical secrets in cultural industry, become a constraint. This has also aggravated the complexity of technical secret protection in the cultural industry nowadays, which has become a bottleneck for the development of cultural industry. Therefore, how to establish an effective technical secret protection system has become a practical problem that the cultural industry needs to solve urgently.

Synergy, as a form of internal governance in an ecosystem, can effectively avoid internal vicious competition, form external economic benefits and shape win-win thinking in the overall ecosystem [5, 6]. Cooperative relationships, as an externalized form of the synergistic concept, tend to occur between upstream and downstream firms within industrial clusters because they have natural complementary characteristics and tend to adopt proximity to select collaborative relationships [7]. In the cultural industry cluster, due to the dual attributes of both material and spiritual cultural industries, their ideologies and values are objectively similar [8], the degree of closeness between enterprises is higher. Collaboration, as a kind of competition between enterprises seeking long-term interests, will naturally be affected by regional policy orientation [9], synergistic costs [10], synergistic interests [11] and other objective factors, but the essence of the factor also depends on whether the parties to the collaboration have a common vision of collaboration. Technical secrets as a strategic resource that enterprises in the cultural industry cluster urgently need to protect. At the same time, the humanistic uniqueness and integration of technical secrets in the cultural industry itself, which will further deepen the affinity between enterprises that share a common vision, prompt the possibility of inter-enterprise synergy. Therefore, the technical secrets in the cultural industry cluster have the possibility of cooperative protection.

As a more extensive research method, evolutionary game theory has become a heated tool for many scholars to study the collaboration and competition relationship under different fields Various game models were built based on this theory. For example, Khan [12] used a game model to analyze the collaboration and competition between recyclers and remanufacturers, and it was found that IoT technology and reverse supply chain play an important role in the decision-making process of the enterprise. Based on a game model of evolutionary cooperation between enterprises in the supply and demand network, it was found that the strategic choices of enterprise cooperation and competition would be unstable [13]. The reason behind is that the evolutionary game theory is consistent with the simulated situation that decision maker has conflicts in cooperation and competition under the objective environment of multi-factor influence. This is more consistent with the imperfectly rational behavior of the decision maker, which is based on limited rationality, and which influences the shift of decision-making mode when facing the conflict of interest of other decision-makers [14]. Therefore, from the perspective of evolutionary game, in the collaborative and non-collaborative protection relationship between enterprises in the cultural industry cluster, uncooperative protection is a self-centered or weakly interactive protection mode, a relatively closed "island mode" [15]. Collaborative protection, on the other hand, can break through the limitations of single-subject governance, and has better results in resource allocation and governance efficiency [16, 17], which can reduce vicious competition, prevent opportunistic leakage of technological secrets, and increase the overall cluster benefits, but at the expense of additional synergistic costs and the prevention of trust crises. In addition, the public sector, such as the government, as the external environment governance body of the cultural industry cluster, to a certain extent, is involved in the synergistic protection issues among enterprises, more in the establishment and protection of public value [18], but its behavior is difficult to solve the social problems arising from the competition between enterprises with each other at the root of the problem. The government and other public sectors, because of its own authority and public nature, can build a contractual bridge of trust between the collaborative subjects, and enterprises within the cultural industry cluster can participate in the mutual game of collaborative protection on their own. Its essence is that in the spirit of contract and the mechanism of government regulation, each participating subject, in order to ensure the maximization of their own interests, constitute a mutual game with other enterprises [19, 20]. This game has the potential to develop into a stable and win-win dynamic collaborative protection model, which can be deduced using an evolutionary game model.

However, existing research focusing on the synergistic protection of technical secrets within cultural industry clusters is relatively lacking, while focus on the protection of technical secrets for industrial clusters mainly in the aspects of industrial policy [20], management mode [15, 21], and mechanism factors [22], and have not been studied in combination with the uniqueness and synergy of cultural industry clusters, which makes the research conclusions lack pertinence. Moreover, the decision-making of enterprise decision-makers is a comprehensive decision-making in a dynamic environment, not only unilaterally consider the static and ideal factors researched by the above scholars, but also more dynamic and comprehensively combine the intrinsic and extrinsic factors, and has not yet introduced the evolutionary game theory to analyze the possibility of synergistic protection under the technological secrecy in the research methodology, which proposes that the main body’s strategy in the system is the result of the enterprise under the assumption of limited rationality. It is proposed that the subject strategy in the system is the result of the enterprise under the assumption of limited rationality. In order to resolve the paradoxical issue of the cooperative protection of technical secrets within the cultural industry clusters, this paper combines the policy background and enterprise management practice of China’s cultural industry via a B2B2G (Business to Business to Government) model of synergistic protection of technical secrets based on the synergistic mechanism of the elemental system within the cultural industry. The government is chosen in this study to act as a trust contract bridge of cooperation to assure the potential of synergistic beginnings because there is a shortage of trust foundation platform in transaction and cooperation as a result of the asymmetric natural factor of information in firms [23]. Also, this paper selects another theoretical perspective of evolutionary game to illustrate the collaborative protection strategy of cultural industry cluster technical secrets and to explore the possibility of the collaborative protection of technical secrets within the cultural industry cluster and the key factors affecting the choice of its strategy. In the evolutionary game theory, the interests are taken as the driving factor of collaborative protection [24], while leading enterprises and following enterprises are deemed as the game main body with limited rationality. By studying the major variables influencing the development of the cooperative protection strategy of the game subjects through the use of data simulation, this paper hopes to draw conclusions and provide recommendations for the cooperative protection of technical secrets in the future.

## 2. Literature review

### 2.1 Evolutionary game theory

According to traditional game theory, people are "fully rational" decision-makers. However, decision-makers are often limited by their own cognition and environmental information differences, so game participants can only make satisfactory rather than optimal decisions [25]. Due to the bounded rationality, evolutionary game theory views the adjustment of group behavior as a dynamic process, which breaks through the limitations of the assumption of complete rationality and emphasizes dynamic equilibrium [26]. Under this context, three implications are included in the game theory: the two parties of the game may fail to reach a Nash equilibrium point; the game process means to gradually reach the equilibrium point; and both parties may find it difficult to reach the optimal equilibrium point [27].This means that the game decisions are not made by just one side. Only after continuous revision and adjustment, can satisfactory results be achieved [28]. In addition, evolutionary game theory is based on the game payoff matrix model. It means that each game participant learns and updates game strategies according to the designed evolutionary rules. There are mainly three steps: first, the game payoff or fitness is calculated, to obtain an expected return on the game process [29]. Second, similar partners are selected to compare payoffs and decide whether to imitate the other’s strategy [30]. Third, knowledge storage and cognitive processes are learnt or updated, which affect the decision-making process of changing cooperation partners [31]. The game process is basically in line with the assumption of finite rational human in real-life decision making and is consistent with the fact that different subjects develop different levels of sensitivity to different influencing factors and will adjust their game strategies according to their own interests and the opinions of different parties [32]. Therefore, introducing evolutionary game theory into group behavior decision-making can better analyze the costs and benefits required by both parties of the game when making decisions in complex and uncertain environments [33].

### 2.2 Research on the protection methods of technical secrets within the cultural industry cluster

Technology secrets are an important asset of the cultural industry, so a good industrial environment needs to be established and opportunistic behavior should be avoided, scholars at home and abroad have conducted extensive research on the protection. Although there is a certain degree of difference among scholars on the protection methods, they generally discuss the methods of punitive outside environment and closed inside environment.

Highly punitive deterrents are adopted through legislation in the external environment to curb opportunistic malpractices. In 2016, the Trade Secrets Protection Act (DTSA) in the United States stipulated that fines for theft of trade secrets by organizations were raised to $5 million, In 2018, China’s Anti-Unfair Competition Law also raised the penalty for theft of trade secrets to $3 million. However, this still can’t avoid the theft and disputes brought about by the economic value attribute of technical secrets. In the legal protection of technical secrets, although the mandatory technical secrets will be supported by external law, but its secret is difficult to determine and evidence [34], the judicial defense of technical secrets is difficult and long. Or, if the commercial license of technical secrets is difficult to obtain, it is also easy to induce the free-riding behavior of potential licensors [35]. In essence, the legal protection of the right to defend is only a bottom-up project, which cannot accurately and effectively meet the timely interests of the technical secret rights holders. On the other hand, some scholars have begun to formulate relevant approaches to protect the interests of rights holders from the source of production of technical secrets. Due to the technical secret information needs to be based on the storage of physical carrier, will be because of the flow of talent and mobility, mastery of technical secrets of senior technical and managerial personnel and then inauguration is bound to increase the likelihood of disclosure of technical secrets. The internal management of technical secret protection focuses on the source of dissemination, dissemination channels, and information reception in three aspects, that is, the focus lies on the three key management links of confidential personnel, technical carriers, and external exchanges [36]. For an enterprise, the biggest threat to technical secrets is from current or former employees [37], Deng [38] proposes to adopt the signing of non-compete contract for the personnel involved in confidentiality, which requires that during the period of employment or in a certain period or area after leaving the job, they shall not operate, be employed or operate the same or similar business activities as theirs. Alternatively, in the TCM cultural industry, screening for oral teaching and master-apprentice teaching in the areas of formulation, production, and processing techniques, diagnosis, and treatment [39]. As for the dissemination channels and information reception, Pedraza-Farina [40] proposes to control the use of technical secrets according to the characteristics of the industry and narrow the informal knowledge and information sharing network. Or to limit the channels of technical secret protection circulation by means of new, not yet widely disseminated encryption technology [41]. Although these means of protection curb the leakage of technical secrets to a certain extent, they cannot avoid informal learning and cooperation across firms, challenging the over-privatization of technical secrets and self-help protection systems [40].

In summary, the above scholars’ views are based on internal and external defense to protect technical secrets from loss, but "blockage is better than sparse", a small number of scholars have begun to adopt cooperative opening strategies to protect technical secrets. Based on the cultural industry cluster technical secrets widely exist in different departments, technology development stage, can be transformed into a small-scale dissemination of information cooperation resources [42]. Alternatively, there may be multiple enterprises within the industry that are the attributors of technical secrets in the competitive relationship, and the construction of a symbiotic system between enterprises can bring into play the most efficient reciprocal symbiosis of technical secrets [43]. At the same time, factors such as geographic proximity within the cultural industry, which has a facilitating role in shaping the exchange of ideas and collaboration [44], and the basis of project cooperation within the industry and the vision of making it bigger and stronger can provide an effective opportunity for synergistic protection of technical secrets.

### 2.3 Influencing factors of technology secret collaborative protection within cultural industry clusters

The competitive advantage of enterprises comes from the cooperation between enterprises and other enterprises [7]. As a complex ecosystem with numerous environmental actors, cultural industry clusters are essentially an environmental behavior based on internal and external factors [21]. Environmental behavior is the common result of a series of interrelated internal and external factors, including external factors of government policy, social system; Internal factors include economic incentives, information intentions, etc [45].

From the study of external factors, scholars believe that industrial policy is the primary factor that induces collaborative conservation. Eastin [20] through a study of several different administrative regions, found that multi-party collaborative formulation of industrial policy can promote collaborative maintenance of industrial environments and maintain a stable cooperative environment. The policy support of the national industry, the more it can also reflect the multi-level and multi-directional synergistic protection of cultural heritage industries [9]. Moreover, if the government can adopt additional subsidy funding policy, it will further promote the positive synergy among the participating members [46]. In terms of internal factors, as a means of integrating complementary resources, the primary goal of synergy is to achieve benefits that outweigh the value of the costs incurred. When the cost of synergy exceeds the benefits gained, it is often easy to dampen the willingness of participating subjects to synergize [10]. The increase in the benefits of collaborative innovation further stimulates the maintenance of effective collaborative relationships [11]. Moreover, along with the expansion of the collaboration scale of cultural enterprises, the diminishing marginal cost drives the willingness of long-term cooperation [47]. In addition, since the information willingness of firms during collaboration is not identical, what they pursue is not a single visible benefit. If in a strong contractually constrained governance model, firms tend to prefer opportunistic risk reduction, which will have a positive impact on participation in collaborative alliance stabilization [48]. Alternatively, firms will choose collaborative strategies to meet the cost reduction of eliminating production technology differences and maintaining long-term cooperation [49]. At the same time, some enterprises in order to get learning in management information, the adoption of synergistic strategy can make their own dominant industry and non-dominant industry to produce the best evolutionary stabilization strategy, but also to achieve synergistic profit increase, synergistic costs become smaller possible [50].

## 3. Construction of cultural industry cluster technology secret evolutionary game model

Since the cultural industry cluster is composed of many interrelated enterprises, the group behavior of these enterprises determines the way to protect the technical secrets of the cultural industry cluster. The cooperation between enterprises is to obtain the market advantage or benefit scale that a single enterprise does not have, but such advantage must be based on mutual trust and for the purpose of mutual benefit [43]. Therefore, the presently constructed collaborative protection game model based on the trust of authoritative government and contractual spirit for the purpose of mutual benefit is in line with the requirements of cooperation. Due to information asymmetry, leading enterprises in the cultural industry cluster have a relative advantage in terms of the volume of technological secrets and the scope of benefits, but there is also a risk of technological innovation spillover and employee mobility. Following enterprises are at a disadvantage when it comes to information, having fewer technological secrets and a smaller enterprise scale, but their employees are generally reliable and have strong imitation motives. In the cultural industry cluster, the leading enterprises, and following enterprises share a lot of knowledge, have mutual mobility of personnel, and have similar technical secrets. These factors are accompanied by either mutual competition or cooperation. As a result, the subject of the game based on the government’s credibility to create a foundation of trust and cooperation, the two sides of the collaboration contract, the subject of the leakage of technical secrets into the scope of the contract for collaborative protection, can significantly limit the flow of technical secrets leakage. While the technical secrets involved in the staff will be restricted to flow into the contract alliance, to avoid reciprocal vicious competition, the contract subject can share the technical secrets within the scope of the contract between the two sides. This will consciously protect the contract within the technical secrets from leakage. Meanwhile, if both parties find that the coordinated protection of technical secrets is more profitable than non- collaborative protection in the first round of the game, they can continue the coordinated protection in the next round; similarly, when the profit of coordinated protection of technical secrets is lower than the profit of non-collaborative protection, both parties can choose to withdraw from coordinated protection in the next round and choose to protect their technical secrets independently. This decision process is a dynamic one and involves various changes, the leading enterprises in the cultural industry cluster and the following enterprises in the technical secret protection of the decision constantly change, and finally reach an equilibrium state.

### 3.1 Model assumptions

**Hypothesis 1:** The leading enterprises and following enterprises are the major participants in a fully developed cultural industry cluster. Bounded rationality allows both parties to maximize their own interests by tailoring their selection tactics to each game round. Leading enterprises and following enterprises have two strategic choices, namely collaborative protection of technical secrets and uncollaborative protection of technical secrets. Collaborative protection is the joint protection of technical secrets, through the contract to master the diffusion of knowledge and control the diffusion of the object, to grasp the industrial ecological right to speak and take the initiative. The uncollaborative protection is to keep the original defective self-protection form and maintain the exclusive advantage of the technology. The value realization of diverse assets can be quantitatively represented by examining the inputs and returns of all assets of an enterprise, and the value realization of intangible assets of a corporation (such as trademarks, technical secrets, etc.) can also be elaborated [51]. The total number of technological secrets can thus replace the entire value included in the enterprise’s technical secrets, which is useful for the model’s intuitive data. This is because the input and return analysis of technical secrets can quantify the economic value it contains. At the same time, leading enterprises have advantages over information, assuming that ***A1***, ***A2*** are the technical secrets owned by leading enterprises and following enterprises respectively, where ***A1***>***A2***.
**Hypothesis 2:** The probability of leading enterprises choosing to participate in collaborative protection technology secret activities is ***x***, and the probability of choosing uncollaborative protection technology secret is ***(1-x)***; the probability of cluster following enterprises to choose collaborative protection is ***y***, and the probability of choosing uncollaborative protection technology secret is ***(1-y)***, where x and y are functions of time t and both are between [0,1].
**Hypothesis 3:** Both leading and following enterprises within the cultural industry cluster choose the strategy of synergistic protection, as the heterogeneous class of distinctive cultural resources within the industry, which contains a large cultural knowledge creativity and high industrial innovation, is prone to bursting into cultural joint economic consumption [4]. It is assumed that technical secret knowledge synergy will produce synergistic benefits ***d*** (such as intellectual property linkage effect, which can better resist external infringement and bring additional knowledge collision effect), and when enterprises carry out synergistic protection, technical secrets will be transferred and exchanged internally, and based on information asymmetry, the absorption and integration coefficients of the leading enterprise and the followers’ enterprise to the other party’s technical secrets are ***B1*** and ***B2***, respectively, and ***B1***> ***B2***.
**Hypothesis 4:** The government plays the role of regulating and assisting in the protection of technical secrets, encouraging the collaborative protection of technical secrets among enterprises and supporting the collaborative development within the cultural industry alliance, and governments at all levels begin to implement different helping policies for enterprises within the cultural industry cluster [52], assuming that each item of the subsidy for the collaborative protection of technical secrets is given with a subsidy coefficient ***S***.
**Hypothesis 5:** In order to avoid the risk of opportunism caused by the incompleteness of the contract, one party adopts synergistic protection and the other party does not adopt synergistic protection, which induces "free-riding" behavior [35], assuming that the additional benefit of free-riding to the leading firm and the followers is ***N1***,***N2***, respectively, so in order to avoid "free-riding", the party that does not synergize with the protection of the confiscation of collaborative deposits T to compensate for synergistic protection of the party that is brought by the loss of "free-riding". According to the above assumptions, we can get the income matrix of leading enterprises and following enterprises, as shown in Table 1.

**Table 1:** Cultural industry cluster game both parties of intellectual property protection collaborative and non-collaborative payment matrix.

Both parties of the game		Following enterprise	
		collaborative protection	uncollaborative protection
Leading enterprise	collaborative protection	R1+A2B1+A1S+dA1/(A1+A2)-C1, R2+A1B2+A2S+dA2/(A1+A2)-C2	R1+A1S+T−C1,R2+N2−T
	uncollaborative protection	R1+N1−T,R2+A2S+T−C2	R1, R2

***R1*** and ***R2*** in the table are the income obtained by leading enterprises and following enterprises to independently protect technical secrets; ***C1*** and ***C2*** are the cost of collaborative protection of technical secrets for leading enterprises and following enterprises, namely, maintenance cost; ***dA1/(A1+A2)*** and ***dA2/(A1+A2)*** are the collaborative profits gained by the collaborative protection of Leading enterprises and following enterprises respectively.

#### 3.2 Local stability analysis

According to the payoff matrix in Table 1, the expected revenue of leading enterprises’ collaborative protection of technical secrets is:

E1n=y[R1+A2B1+A1S+dA1/(A1+A2)−C1]+(1−y)(R1+A1S+T−C1) (1)

The expected revenue of leading enterprises adopting uncollaborative protection of technical secrets is as follows:

E1m=y(R1+N1−T)+(1−y)R1 (2)

The average revenue of leading enterprises is:

E1=xE1n+(1−x)E1m (3)

The replicator dynamic equation for constructing the decision selection of leading enterprise is:

$$F(x)=\frac{dx}{dt}=x(E1n-E1)=x(1-x)(E1n-E1m)=x(1-x)[y\left(\frac{dA1}{A1+A2}+A2B1-N1\right)+A1S+T-C1]$$ (4)

Similarly, the expected revenue of following enterprises in the cultural industry cluster to adopt collaborative protection of technical secrets is as below:

E2n=x[R2+A1B2+A2S+dA2/(A1+A2)−C2]+(1−x)(R2+A2S+T−C2) (5)

The expected revenue of following enterprises adopting uncollaborative protection of technical secrets is as follows:

E2m=x(R2+A1B2−T)+(1−x)R2 (6)

The average revenue of following enterprises in cultural industry cluster is:

E2=yE2n+(1−y)E2m (7)

The replicator dynamic equation set of following enterprises’ decision choice in cultural industry cluster is:

$$F(y)=\frac{dy}{dt}=y(E2n-E2)=y(1-y)(E2n-E2m)=y(1-y)[x\left(\frac{dA2}{A1+A2}+A1B2-N2\right)+A2S+T-C2]$$ (8)

The evolution of enterprise decision-making selection is carried out by replicator dynamic Eqs (4) and (8). Let F(x) = 0 and F(y) = 0, respectively, from which the plane m={(x,y)|0≤x,y≤1}can be obtained. If $0<x*=\frac{C2-A2S-T}{\frac{dA2}{A1+A2}+A1B2-N2},y*=\frac{C1-A1S-T}{\frac{dA1}{A1+A2}+A2B1-N1}<1$, there are five equilibrium points: (0, 0), (1, 0), (1,1). (x *, y *), otherwise there are four equilibrium points: (0, 0), (1, 0), (0, 1), (1,1).

According to the method proposed by Friedman [53], for a group dynamic system described by differential equations, the stability of the evolutionary system can be judged by the local stability analysis of the Jacobian matrix. The Jacobian matrix of the above model is:

$$J=\left[\begin{array}{cc} \frac{\partial F(x)}{\partial x} & \frac{\partial F(x)}{\partial y}\\ \frac{\partial F(y)}{\partial x} & \frac{\partial F(y)}{\partial y} \end{array}\right]=\left[\begin{array}{cc} \left(1-2x)[y(\frac{dA1}{A1+A2}+A2B1-N1)+A1S+T-C1\right] & x(1-x)(\frac{dA1}{A1+A2}+A2B1-N1)\\ y(1-y)(\frac{dA2}{A1+A2}+A1B2-N2) & \left(1-2y)[x(\frac{dA2}{A1+A2}+A1B2-N2)+A2S+T-C2\right] \end{array}\right]$$ (9)

The determinant of the Jacobian matrix:

$$Det(J)=(1-2x)\left[y(\frac{dA1}{A1+A2}+A2B1-N1)+A1S+T-C1\right](1-2y)\left[x(\frac{dA2}{A1+A2}+A1B2-N2)+A2S+T-C2\right]$$ (10)

The trace of the Jacobian matrix is:

$$Tr(J)=(1-2x)\left[y(\frac{dA1}{A1+A2}+A2B1-N1)+A1S+T-C1\right]+(1-2y)\left[x(\frac{dA2}{A1+A2}+A1B2-N2)+A2S+T-C2\right]$$ (11)

According to the local analysis method of matrix, the local stability analysis table of evolutionary game is obtained, as shown in Table 2.

**Table 2:** Local stability analysis table of evolutionary game of enterprise technology secret protection strategy in cultural industry cluster.

Point of Equilibrium	Det(J)	Tr(J)
O (0,0)	(A1S+T−C1)(A2S+T−C2)	A1S+A2S+2T−C1−C2
A (0,1)	$-\left(\frac{dA1}{A1+A2}+A2B1-N1+A1S+T-C1\right)(A2S+T-C2)$	$\left(\frac{dA1}{A1+A2}+A2B1-N1\right)+A1S+C2-A2S-C1$
B (1,0)	$-(A1S+T-C1)(\frac{dA2}{A1+A2}+A2S+T-C2)$	$(\frac{dA2}{A1+A2}+A1B2-N2)+A2S+C1-A1S-C2$
C (1,1)	$\left[(\frac{dA1}{A1+A2}+A2B1-N1)+A1S+T-C1\right]\left[(\frac{dA2}{A1+A2}+A1B2-N2+A2B1-N1)+A2S+T-C2\right]$	$-\left[(\frac{dA1}{A1+A2}+A2B1-N1)+(\frac{dA2}{A1+A2}+A2B1-N1)+A1S+T-C1+A2S+T-C2\right]$
D (x*, y*)	0	0

The game subject as a limited rationality of the subject, without considering the impact of its policy and other external factors, the comprehensive benefits as the willingness to collaborate to protect the first condition, in which, due to the hitchhiking belongs to the stage behavior, the comprehensive hitchhiking brought about by the benefits is to be less than the comprehensive benefits of collaborative cooperation, that is, dA1/(A1+A2)+A2B1>N1,dA2/(A1+A2)+A1B2>N2, according to the local analysis in Table 1, it is found that the stability analysis of the five equilibrium points can be carried out directly by discussing the different parameter values of A1S+T≶C1,A2S+T≶C2, and, at the same time, determining whether ***Det(J)>0*** and ***Tr(J)<0*** are satisfied, so as to determine if the equilibrium point is the evolution of system localized asymptotically stable immobile point in the evolution, i.e., the evolutionary stability strategy [53].

**Fig 1:**
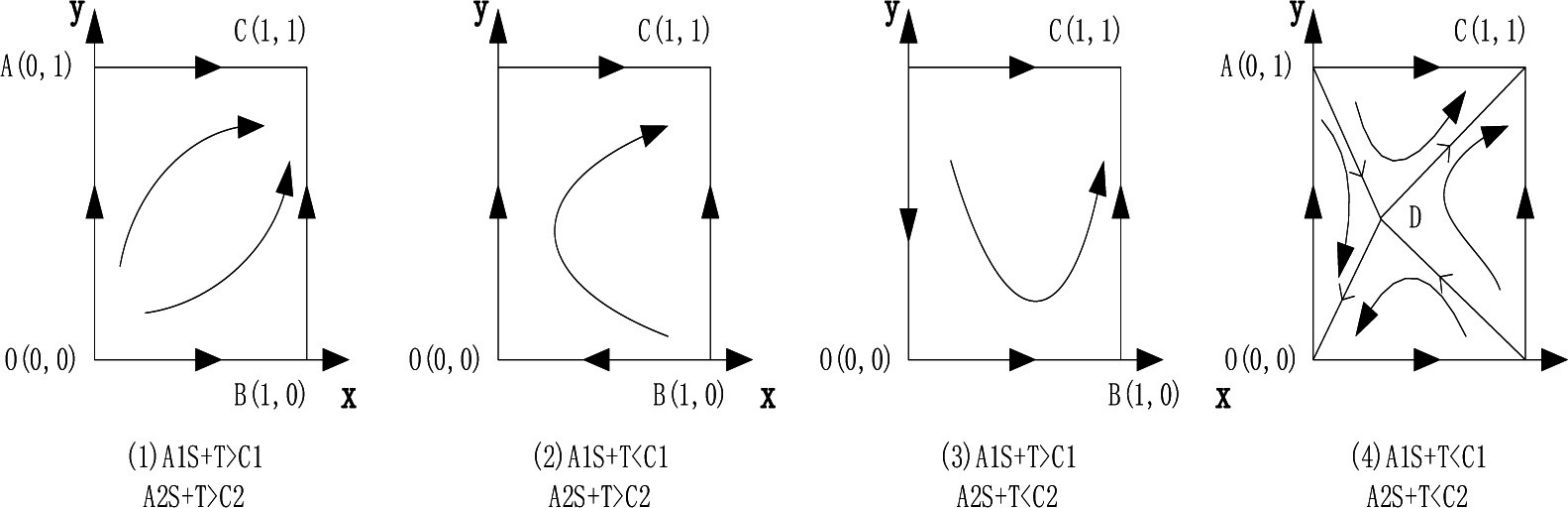
Evolutionary phase diagram of strategy choice between leading enterprises and following enterprises in cultural industry cluster.

(1) In scenario 1, When A1S+T>C1,A2S+T>C2, that is, the sum of government subsidies and cooperative deposit compensation received by the leading enterprises and the following enterprises is greater than their cooperative protection costs, the local stability analysis table is shown in Table 3. there are four equilibrium points (0,0), (1,0), (0,1), (1,1), where C (1,1) is the local stability point, and the phase diagram is shown in Fig 1 (1). In order to protect technical secrets and obtain benefits, leading enterprises and following enterprises are willing to transform technical secrets into common intellectual property rights for collaborative protection, give up exclusive advantages and obtain synergies together. Leading enterprises and following enterprises in cultural industry clusters choose collaborative protection as an evolutionary stability strategy.

**Table 3:** Scene 1 stability analysis table.

Point of Equilibrium	Det(J)(sign)	Tr(J)(sign)	Local stability
O (0,0)	+	+	Unstable point
A (0,1)	-	+, -	Saddle point
B (1,0)	-	+, -	Saddle point
C (1,1)	+	-	ESS

(2) In scenario 2, When A1S+T<C1,A2S+T>C2 or A1S+T>C1,A2S+T<C2, that is, when the sum of government subsidies and cooperative deposit compensation received by one of the leading enterprises and the following enterprises is less than the cooperative protection cost, and the sum of government subsidies and cooperative deposit compensation received by the other party is greater than the cooperative protection cost, there are four equilibrium points (0, 0), (1, 0), (0, 1), (1,1), where C (1,1) is the local stability point. The local stability analysis table is shown in Table 4, and the phase diagrams are shown in Fig 1 (2) and Fig 1 (3). For leading enterprises and following enterprises in cultural industry cluster, the evolutionary stable strategy is to collaborative protection.

**Table 4:** Scene 2 stability analysis table.

A1S+T<C1,A2S+T>C2				A1S+T>C1,A2S+T<C2			
Point of Equilibrium	Det(J) (sign)	Tr(J) (sign)	Local stability	Point of Equilibrium	Det(J) (sign)	Tr(J) (sign)	Local stability
O(0,0)	-	+, -	Saddle point	O(0,0)	-	+, -	Saddle point
A(0,1)	-	+, -	Saddle point	A(0,1)	+	+	Unstable point
B(1,0)	+	+	Unstable point	B(1,0)	-	+, -	Saddle point
C(1,1)	+	-	ESS	C(1,1)	+	-	ESS

(3) In scenario 3, When A1S+T<C1,A2S+T<C2, that is, the sum of government subsidies and cooperative deposit compensation received by the leading enterprise and the following enterprise is less than its cooperative protection cost. There are five equilibrium points (0,0), (1, 0), (0, 1), (1,1), (x*, y*), in which O(0,0) and C(1,1) are local stability points. The local stability analysis table is shown in Table 5. Leading enterprises and following enterprises can not only choose collaborative protection if they want to obtain the government’s technical secret subsidies and collaborative protection benefits. However, at the same time, they will also adopt the independent protection to master their own core technical secrets and occupy the unique advantages of the industry. Leading enterprises and following enterprises in cultural industry clusters can choose collaborative protection or independent protection.

**Table 5:** Scene 3 stability analysis table.

Point of Equilibrium	Det(J) (sign)	Tr(J) (sign)	Local stability
O (0,0)	+	-	ESS
A (0,1)	+	+	Unstable point
B (1,0)	+	+	Unstable point
C (1,1)	+	-	ESS
D (x*, y*)	0	0	Saddle point

Summarizing the local stability analysis of the evolutionary game, the strategy choice of the game subject has a direct relationship with the government subsidy coefficient for each technical secret (***S***), the number of technical secrets provided by the enterprise (***A1*, *A2***), the cost of collaborative protection (***C1*, *C2***), and the collaborative deposits (***T***), in which the cost of collaborative protection serves as a key influencing factor for the game subject. According to A1S+T≶C1,A2S+T≶C2, the game between the two parties of the game can be categorized into two modes. In Mode 1, A1S+T>C1andA2S+T>C2. When the synergistic risk benefit of each party (the sum of the collaborative deposits compensation and government subsidy) is greater than their synergistic protection cost, the changes of other influencing factors cannot prevent the two sides of the game from reaching a synergistic protection consensus. Another situation is A1S+T<C1,A2S+T>C2 or A1S+T>C1,A2S+T<C2. When one side of the game’s technical secret synergistic risk benefit is greater than its synergistic protection cost, the other side of the game’s technical secret synergistic risk benefit is smaller than its synergistic protection cost, both sides of the game will reach a consensus on co-protection as well. In Model 2, A1S+T<C1,A2S+T<C2, the technical secret synergistic risk benefit (the sum of collaborative deposits compensation and government subsidy) is less than their cost of coordinated protection, both sides of the game will begin to diverge in their strategies, and will begin to choose either non-cooperative or coordinated protection strategies according to their interests.

## 4. Experimental data and simulation

**Fig 2:**
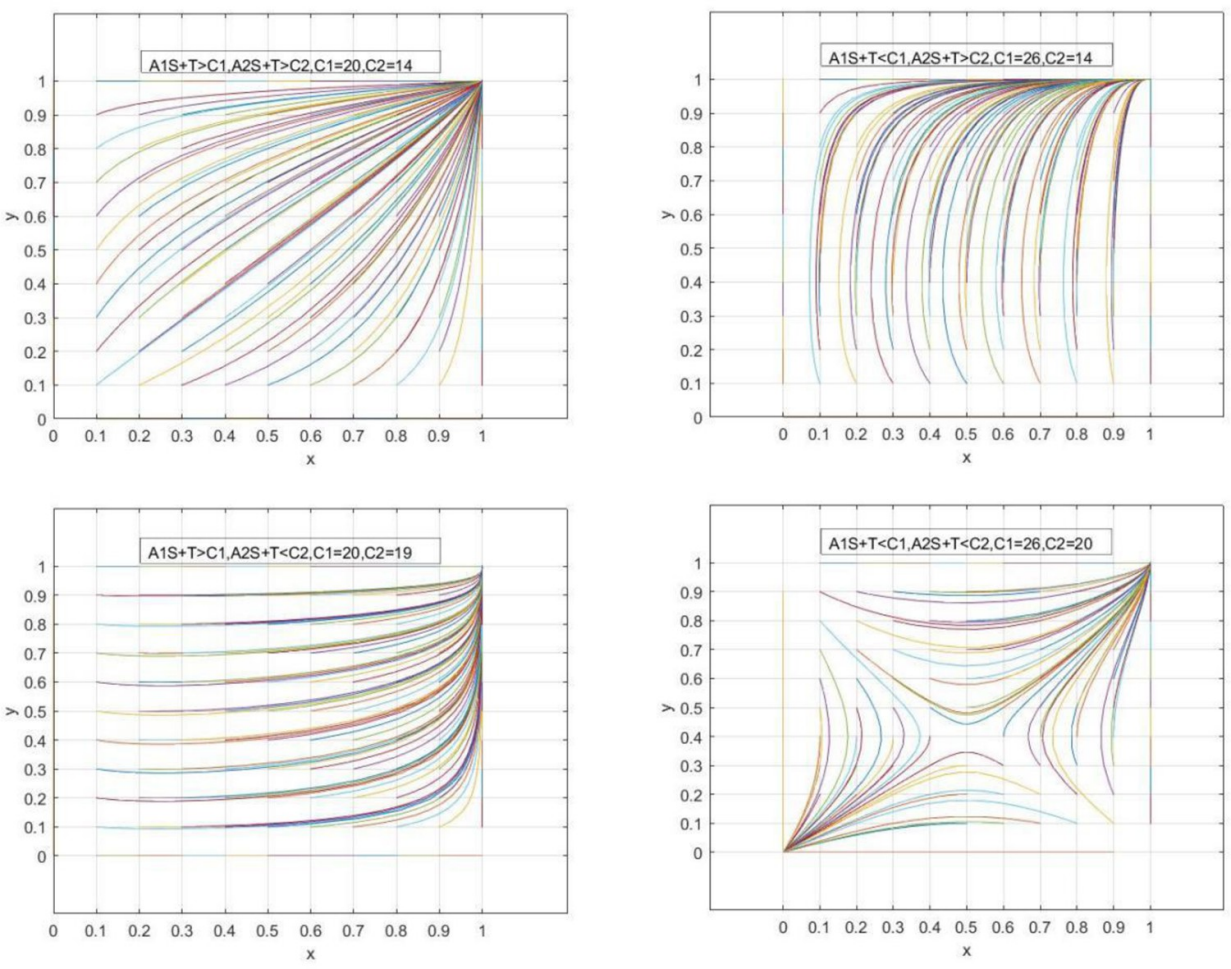
Simulation of replicated dynamic evolutionary game phases with different parameters.

In order to verify the authenticity of the above three scenarios, this study uses MATLAB R2021a software to simulate and analyze each parameter. Although the setting of the initial value of the parameter affects its evolution range and speed to a certain extent, it will not change its overall trend and results [54, 55]. The most important thing is the accuracy of the model establishment and the practicality of the model, which aims to reveal the regular characteristics of things changing [56]. Therefore, based on the requirements of the model, the existing relevant research [57, 58], parameter setting law, analogy [59], research on its assignment, ***A1 = 60*, *A2 = 40*, *B1 = 0*.*3*, *B2 = 0*.*2*, *N1 = 25*, *N2 = 20*, *S = 0*.*3*, *d = 30*, *T = 6*,**, simulation game subject to participate in collaborative protection willingness (***x*, *y***) all values, changes of collaborative protection cost (***C1*, *C2***), and simulation verification of the conditions A1S+T≶C1,A2S+T≶C2, the following simulation results are obtained (Fig 2).

Both sides of the game will start to diverge in their strategies and will start to choose either non-cooperative or coordinated protection strategies depending on their interests because the technical secret synergistic risk benefit (the total of collaborative deposits compensation and government subsidy) is less than their cost of coordinated protection. However, when the cost of synergistic protection exceeded both sides’ expectations of the synergistic risk benefit. As a result, synergistic protection will be weakened as the cost of synergistic protection gradually increases. As a result, when deciding on a game’s strategy, both sides are more willing to bear a synergistic cost rather than the synergistic risk benefit of maintaining technological secrets, which can be considered as a first step in the process. Therefore, when selecting the subject for game strategy, both sides of the game to pay the synergistic cost compared to the synergistic risk benefit of technical secrets can be a preliminary strategy judgment, when A1S+T<C1,A2S+T<C2, the subject of the game strategy begins to be affected by the government subsidies (***S***), collaborative deposits (***T***), synergistic benefits (***d***) and the difference in value of the technical secrets and other related factors.

In order to further investigate the degree of influence of the above analyzed deduced influencing factors on the strategy of the game subject, under the satisfaction of the basic conditions of the study dA1/(A1+A2)+A2B1>N1,dA2/(A1+A2)+A1B2>N2, in Model 2, the relevant factors such as government subsidy (***S***), collaborative deposits (***T***), synergistic benefit (***d***) and technological secret differentials will be sensitivity measurements to explore those factors that have a stronger influence on the synergistic protection strategy. Therefore, the control variable method is adopted to simulate the changes of the above factors within the feasible range. Since the benefits obtained by leading enterprises in participating in collaborative protection and the distress of technical secret protection are relatively more with following enterprises, their willingness to participate in collaborative protection is higher. Therefore, in order to facilitate the observation of the relevant factors affecting the strategy change of the main body of the game, the initial values of ***x*** and ***y*** are set to 0.6 and 0.4 in the following game.

### 4.1 Impact of government subsidies on evolutionary paths

**Fig 3:**
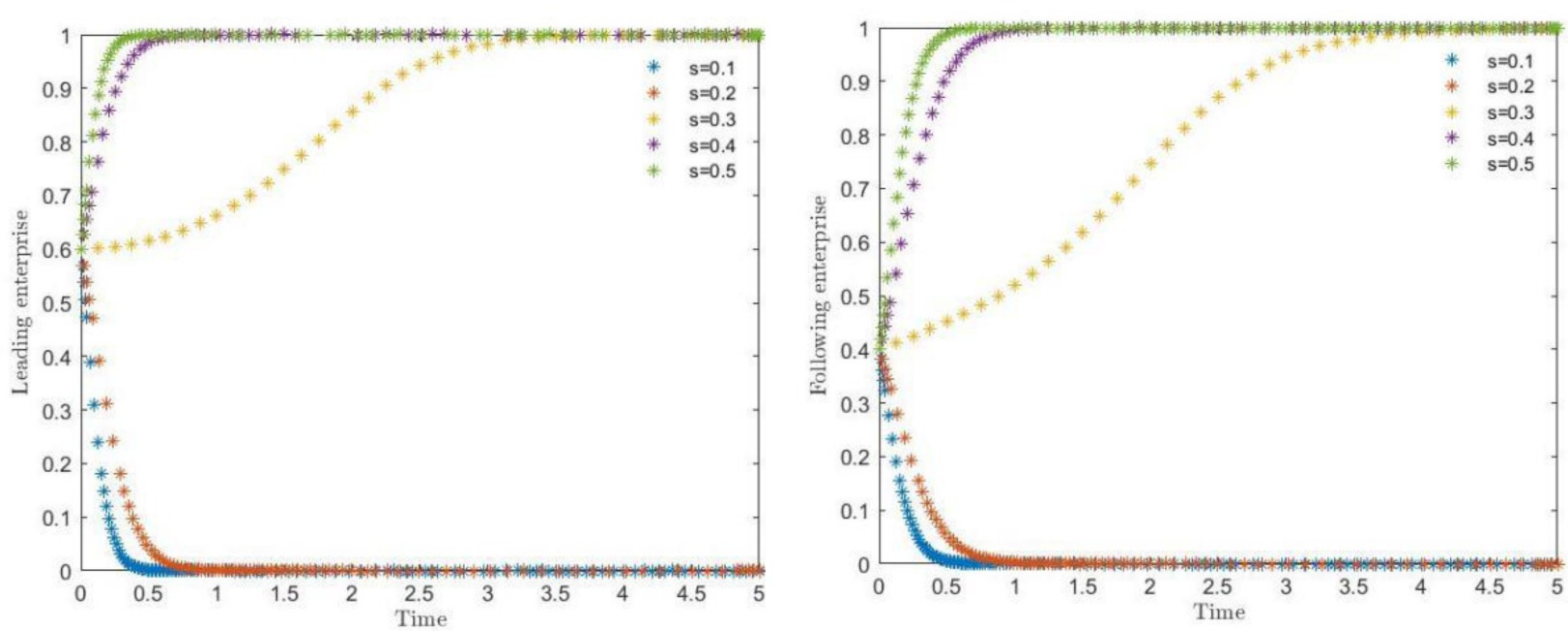
Strategic impacts of government subsidies on leading enterprises and following enterprises.

The simulation results of the effect of government subsidy (***S***) on the evolution of the system are shown in Fig 3. Where the government subsidy coefficient (***S***) took the value of 0.1, 0.2, 0.3, 0.4, 0.5, respectively, to observe the leading enterprises and followers to choose the strategic choice of synergistic protection, as can be seen in Fig 3, when the subsidy coefficient (***S***) is in the range of 0.2 to 0.3, the impact of government subsidy coefficients on the system is also in the synergistic protection and not synergistic protection of the critical value of the critical value of the critical value of the critical value of the synergistic protection. When the subsidy is higher than the critical value, the strategies of both sides of the game begin to tilt to the synergistic protection, and vice versa tends to be unsynergistic protection. In order to encourage the collaboration of cultural industry enterprises, the government gives subsidy policy, although from the parameter setting on the lower value, but can be found from the figure that both sides of the game subject for the government subsidy coefficient rise are more sensitive. From the observation of the convergence speed of the strategies of the two sides of the game, along with the increase of the government subsidy coefficient, due to the leading enterprises involved in the collaborative protection of a larger number of technical secrets, the subsidy gained by them is more, and their speed of convergence is faster than that of the following enterprises. Therefore, increasing the government subsidy coefficient in general is conducive to promoting the role of synergistic protection of both subjects of the game. According to related studies, Zhu et al. [60] based on a sample of 50 culture and media listed companies listed on the Shenzhen and Shanghai stock exchanges from 2013 to 2018, found that government subsidies bring favorable signals to culture industry synergies in the early period and a period of time in the future. Based on the signaling theory, Mattia et al. [61] argued that government subsidies are conducive for enterprises to carry out cooperation with other firms and have a significant impact on the performance of large firms. The findings of their studies all recognize the facilitating role of government subsidies.

### 4.2 Impact of collaborative deposits on evolutionary paths

**Fig 4:**
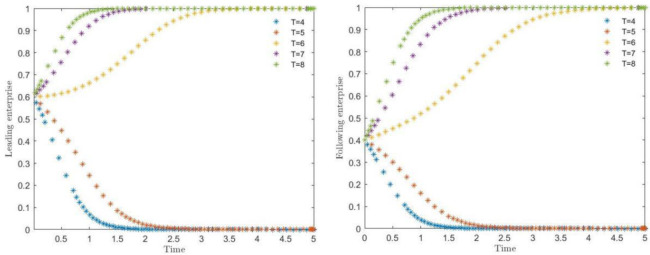
The strategic impact of collaborative deposits on leading and following enterprises.

The simulation results of the effect of collaborative deposits (***T***) on the evolution of the system are shown in Fig 4. Where the collaborative deposits (***T***) took the value of 4, 5, 6, 7 and 8, respectively, to observe the strategy choice of the leading and following enterprises to choose synergistic protection, respectively, as can be seen in Fig 4, when the collaborative deposits (***T***) to between 5 and 6, the strategy of the main body of the game began to shift from the initial non-synergistic protection to in synergistic protection, and with the increasing collaborative deposits, the synergistic protection tendency became significantly accelerated. In order to resist hitchhiking and other unpleasant behaviors, the contractual cooperative deposit set in advance, although from the parameter setting on the low value, for both sides of the game to obtain the benefit of a negligible proportion, but from the figure can be found in the game subject both sides of the cooperative deposit rise for the more sensitive. Contract cooperative deposit increase the two sides of the willingness to cooperate to protect also gradually accelerated, therefore, the increase of cooperative deposit can stabilize the cooperative relationship, can be in the game on both sides of the psychology of the willingness to obtain stable collaboration, is the cooperative contract of the bottom of the pocket. Although the security means can be considered as not the core key factor to drive the willingness of both parties of the game to collaborate, it is indispensable for the stabilization of the whole collaborative protection system. Based on relevant research findings, Chen [62] argues that members involved in economic activities have fairness preferences, and that performance deposits, as a form of pecuniary guarantee, have both compensatory and punitive roles, which can urge the effective implementation of the contract and make up for the excessive harm brought by breach of contract. Yu and Ren [63], based on the study of financing cooperation in China’s cultural industry, found that drawing on the copyright pledge loan method of small and medium-sized enterprises, and flexibly adopting a variety of intangible assets for pooled pledges, can enhance the trust and financial cooperation between financial institutions and cultural enterprises.

### 4.3 Impact of synergies on evolutionary pathways

**Fig 5:**
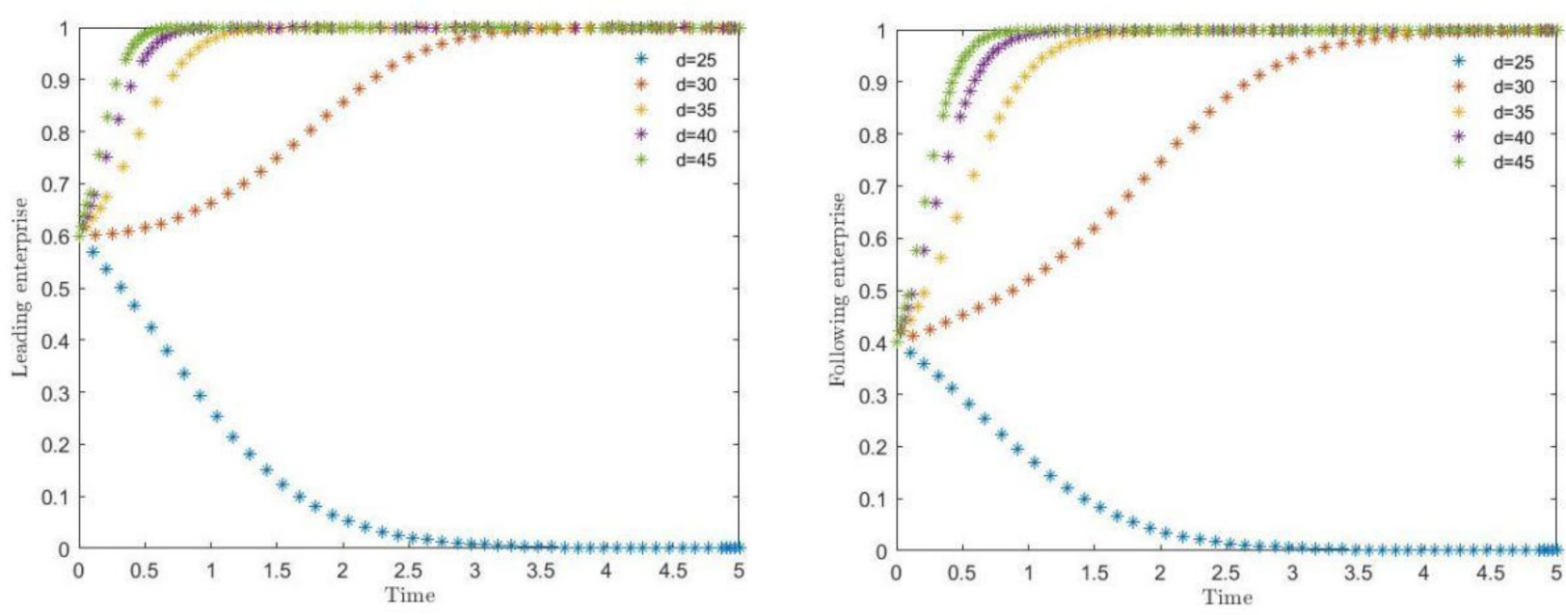
Strategic impact of synergy on leading and following enterprises.

The simulation results of the effect of synergistic benefit (d) on the evolution of the system are shown in Fig 5. Where the synergistic benefit (d) takes the values of 25, 30, 35, 40 and 45, it can respectively observe the strategy selection of leading and following enterprises to choose synergistic protection. When the critical value of synergistic benefit (d) is between 25 and 30, the strategy of the main body of the game begins to choose to shift, and along with the larger synergistic benefit, the trend of both sides of the game to choose the synergistic protection is accelerated, and the time to converge the result begins to reduce substantially. Among them, it can be found that in the synergistic benefit is less than 35, the unit time of convergence between the two sides of the game is obviously longer than the time spent in the synergistic benefit is greater than 35, which also indicates that when the synergistic benefit reaches the situation that the two sides of the game are more satisfied, it will substantially reduce the time of synergistic consideration and promote the rapid conclusion of the contract.

### 4.4 The effect of the difference in technological secrets between the two sides of the game subjects on the evolutionary paths

**Fig 6:**
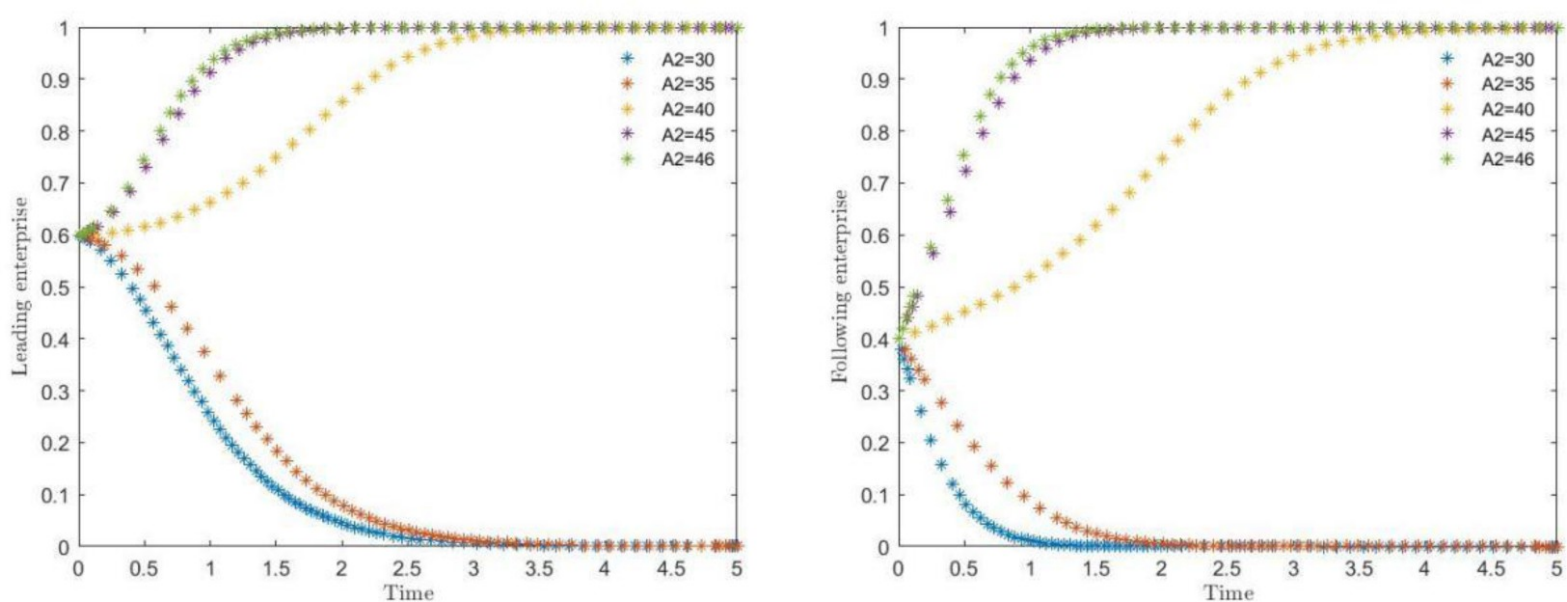
Impact of the difference in the number of technical secrets on the strategies of leading enterprises and following enterprises.

The simulation results of the impact of the difference in the number of technical secrets between the leading and following enterprises on the evolution of the system are shown in Fig 6. Where (***A2)*** respectively take the value of 30, 35, 40, 45, 46, corresponding to the difference in the number of technical secrets of 30, 25, 20, 15, 14 respectively, to observe the leading enterprises and followers to choose collaborative protection of the strategic choice. As can be seen from Fig 6, when the difference in the number of technical secrets between 20 and 15, the two sides of the game subject strategy began to change, and with the smaller the difference, the trend of its game strategy change synergistic protection accelerated. From the game both sides of the strategy convergence speed observation, in the same number of difference before the convergence speed of the leading enterprises faster than the following enterprises, although the leading enterprises hold a large number of industrial technology secrets, follow the enterprise has a small number of technical secrets, to take synergistic protection of the following enterprises will absorb a large number of technical secrets of the leading enterprises, the leading enterprises will absorb a small number of technical secrets of the following enterprises, but the leading enterprises are more inclined to the synergistic protection strategy. Among them, it can also be found that the relevant capabilities and capital of both parties are at similar levels are more likely to achieve the better effect of tacit knowledge collaboration [64]. With the gradual decrease of the difference of technical secrets, the faster the willingness of the game subjects to collaborate on protection, therefore, when the assets of intellectual property and other assets owned by the game subjects are close to the same amount, the possibility of cooperation between the two sides will be greater.

## 5. Research conclusions and recommendations

Based on the policies of "cultural self-confidence and self-reliance" and "prosper of cultural undertakings and cultural industries" put forward by the 20th CPC National Congress [65], an evolutionary game model is established for the synergistic protection of technological secrets in the cultural industry cluster. This evolutionary game model aims to further enrich the research on the evolutionary mechanism of synergistic protection and expand the application scenarios of evolutionary game theory. Based on the strategy evolution of game subjects within the cultural industry cluster under different influencing factors, the following conclusions are drawn:

1. In terms of theoretical analysis, based on the different information asymmetry and capital gap between enterprises, enterprises reach two distinct classical states of stability after repeated games. In Mode 1, when the cost of synergistic protection of both sides of the game is less than the benefit of synergistic risk of technical secrets (the sum of collaborative deposits compensation and government subsidy), leading enterprises and following enterprises will tend to choose a less risky and stable strategy of synergistic protection. In another situation, the cost of synergistic protection of both sides of the game is compared with the result of synergistic risk benefit of technical secrets in a different way. Because of the comprehensive interest and the benefit of technical secret absorption, both sides of the game strategy also prefer synergistic protection strategy. In Mode 2, when the cost of synergistic protection of both sides of the game is greater than the benefit of synergistic risk of technical secrets, the game strategy of leading enterprises and following enterprises can be polarized into two situations (collaborative protection, collaborative protection) or (uncollaborative protection, uncollaborative protection) according to their own interests. Secondly, the main factors behind the game strategy mainly include co-protection cost, government subsidy coefficient, collaborative deposits, and the number of enterprises’ technological secret participation. To be specific, the co-protection cost serves as a key factor affecting the game strategy, and the ration of the game subject’s expenditure (co-protection cost) and benefit of synergistic risk of technical secrets (the sum of collaborative deposits compensation and government subsidy) will also affect the different modeling states of game parties.
2. In terms of simulation analysis, government subsidy coefficient, collaborative deposits, synergistic benefits, and the number of technical secrets is found to affect the evolution of game strategies to different degrees. Among them, the game subject is more sensitive to the government subsidy, collaborative deposits and the changes in the number of technological secrets, while less sensitive to the synergistic benefits generated by the two sides of the game. Stimulating government subsidies, on the one hand, can bring effective collaboration [61], while increasing the proportion of collaborative deposits can hinder the collaboration, cause hitchhiking mentality, and solidify the collaborative relationship [62]. As it requires a longer process for the synergy benefits to demonstrate the impact on collaboration protection and it needs a certain time test, but the increase of synergy benefits can foster the willingness to cooperate. In addition, the difference of technical secrets between the two sides of the game can be further extended and the investors of the two sides can participate in the collaboration. When the capital of the two sides of the game is at a similar level, they are more likely to achieve the better results of tacit knowledge collaboration [64]. The cost of collaborative protection is a major factor influencing the many orientations, according to evolutionary game theory’s perspective on the dilemma of collaborative protection of technical secrets.

Based on the results of the above theoretical analysis and simulation analysis, in order to promote the synergistic protection of technical secrets of cultural industry clusters and improve the synergistic and benign development of cultural industry clusters, which puts forward the following relevant suggestions to the government and enterprises.

1. Lowering the cost of collaboration to enhance the willingness of cooperation between enterprises. Theoretical and simulation analysis has revealed that the cost of collaboration is a key factor in the division of participation mode and the willingness to collaborate and protect, so the government should establish relevant institutions and departments to support collaborative cooperation within the cultural cluster, through the policy support, talent, and technical guidance to reduce the cost of collaboration. As for the enterprises themselves, they need to rely on cultural resources and support policies, make innovations in cultural products, cultural services, and cultural experiences, extend the value chain of cultural industry, and improve the return rate of synergy costs.
2. Increasing collaboration deposits to reduce the risk of free-riding. As a protective measure, the collaboration deposit can deter free-riding behavior within the collaboration. The government and enterprises can discuss raising the collaborative deposits in a way that doesn’t put the interests of synergistic protectors at risk, and can increase the confidence of non-synergistic protectors in the contract, and deter opportunistic actions like hitchhiking.
3. Improving policy subsidies to enhance the benefits of collaboration. Government subsidies play a positive role in promoting collaborative cooperation. First, government should take phased subsidy measures to avoid over-reliance of enterprises on subsidies, to indirectly stimulate the willingness of collaborative protection among enterprises. Secondly, enterprises should be guided to progressively transition away from the pursuit of government subsidies and move toward the joint protection of technical secrets. The specific measures include the formulation of effective trade policy, relaxing the approval conditions of financial institutions for the technology research and development funds of cultural enterprises, increasing the public welfare marketing and promotion inputs for cultural industry enterprises.
4. Reducing the gap of collaboration capital to improve the collaboration system. The gap between technical secret capital between enterprises forms the basis of cooperative protection of technical secrets among enterprises in the cultural industry cluster. The closer technical secret capital is between enterprises, the more it can spur cooperation, which is a powerful combination. Therefore, the government needs to provide more support small and medium-sized enterprises, like preferential policies for enterprises with development potential or cultural characteristics within the industrial cluster or those small and medium-sized followers whose products or services are trendy and have market prospects. On the one hand, the government can build a service platform for cultural industry cluster integrating financial, talent and information services to help small and medium-sized enterprises. On the other hand, small and medium-sized enterprises within the cultural industry cluster should also enhance the R&D investment in technical secret knowledge to narrow the gap of collaborative capital. Specifically, it includes increasing capital investment, increasing the cultivation of technical talents, promoting the exchange of talents, increasing, and sharing their own technical secret capital, to facilitate the cooperation with leading enterprises.

The study makes some contributions to theory and management practice. Firstly, it finds that the protection cost and the capital of the collaborating parties have a significant role in regulating the game strategy, thus enhancing the evolutionary mechanism of cooperative protection in theory. At the same time, analyzing the possibility of collaborative protection of technical secrets in cultural industry clusters from the perspective of evolutionary game, which extends the application of evolutionary game theory in collaboration and competition. Secondly, it offers the concept of cooperative protection mode for the preservation of technical secrets in clusters of cultural industries, which broadens the scope of technical secrets’ protection mode diversity. At the same time, it also highlights the major driving forces behind the advancement of collaborative protection, including collaborative expenses, collaborative deposits, and government regulations, enhancing managers’ ability to make timely decisions about collaborative protection plans.

There are still certain limitations in this study. The study only takes into account the potential for game subject collaboration in heterogeneous types of cultural industry clusters; because of this, the enterprises in the cluster are more likely to produce synergistic interests, increasing the possibility of willingness to collaborate; however, the potential for technical secret collaboration between homogeneous cultural enterprises or other types of enterprises has not yet been included in the study’s scope. We will therefore broaden the variety of collaborative objects in the upcoming research, whether it be the potential for cooperation in homogeneous cultural industry clusters or the potential for cooperation in cross-industry collaborative strategy selection and influence mechanisms.

## Supporting information

S1 File (DOCX)
