# Supplementary material for: Cooperative protection of technical secrets in cultural industry cluster-based on evolutionary game model of leading enterprises and following enterprises
Source: PLoS One. 2023 Sep 14;18(9):e0291459. doi: 10.1371/journal.pone.0291459 (PMC10501660; doi:10.1371/journal.pone.0291459)
Supplement: S1 File — (DOCX) [file pone.0291459.s001.docx]

**Dear Editor:**

Thank you for your letter.

The sources of simulation data for the experimental data in this paper are all borrowed and referenced from existing research papers. Although the reference initial values of evolutionary game will affect the evolutionary magnitude and speed to some extent, the overall trend and result of evolution will not be changed. The most important thing is the accuracy of the model establishment and the practicality of the model, which aims to reveal the regular characteristics of things changing [46]. Therefore, drawing on the existing studies Guo [47], Lu [48] and Su [49], appropriate adjustments are made according to the model of this paper. Links to the paper have been added to the end of the references.

**References**

[1] Sterman JD. Business dynamic analysis method: Systematic thinking and modeling of complex world. Beijing: Tsinghua University Press, 2008.
[2] Guo Y, Liu WQ, Zhang CY, Yu WQ. Evolutionary game analysis on copyright protection of audio knowledge payment products. Modern Intelligence, 2022, 42(4): 134-142. DOI: 10.3969/j.issn.1008-0821.2022.04.012.
[3] Lu XM, Zhang BX, Wang J, Li YX. Evolutionary game research on open source ecological knowledge sharing and risk management in cloud computing. Operation and Management, 2022, 31(8): 101-108. DOI: 10.12005/orms.2022.0257.
[4] Su Y, Guo W, Zhang AR. Reverse transfer of tacit knowledge in mergers and acquisitions based on big data technology. Research Management, 2022, 43 (09): 48-57. DOI: 10.19571/j.cnki.1000-2995.2022.09.006. https://kns.cnki.net/kcms2/article/abstract?v=M9N_p6ifsvOC4WVL-eku8t4RkK6Xz-l2MnrpjRmK6H7NDFOVRoGHIFs-VElU7j89en2OgqZDCXFB-Ep6DcrzLiXIM6TRxPd7TKso3mntPLLxOPRx-A8wmsABLdf-RPVG6cQQaN8dWIM=&uniplatform=NZKPT&language=CHS

| Authors | Articles Data |
| --- | --- |
| Guo [47] | S_1_=10、S_2_=4、S_3_=2、C_1_=6、C_2_=5、C_3_=1、C_4_=3、F=5、P=0.4 |
| Lu [48] | K_1_=60,K_2_=55,ΔK_1_=55,ΔK_2_=50,I_1_=30,I_2_=35,C_1_=30,C_2_=32,R_1_=35,R_2_=30,β=0.2,δ=0.3,W=20 |
| Su [49] | u=0.5,s=0.5,K=350,θ=0.5,e=0.6,T=150,a=0.2,C1=50,C2=30,r=0.4 |
